# A Phase I-II multicenter trial with Avelumab plus autologous dendritic cell vaccine in pre-treated mismatch repair-proficient (MSS) metastatic colorectal cancer patients; GEMCAD 1602 study

**DOI:** 10.1007/s00262-022-03283-5

**Published:** 2022-09-09

**Authors:** Marta Español-Rego, Carlos Fernández-Martos, Elena Elez, Carles Foguet, Leire Pedrosa, Nuria Rodríguez, Ana Ruiz-Casado, Estela Pineda, Joan Cid, Raquel Cabezón, Helena Oliveres, Miquel Lozano, Angels Ginés, Angeles García-Criado, Juan Ramon Ayuso, Mario Pagés, Miriam Cuatrecasas, Ferràn Torres, Timothy Thomson, Marta Cascante, Daniel Benítez-Ribas, Joan Maurel

**Affiliations:** 1 Immunology Department, Hospital Clínic, Barcelona, Spain; 2 Medical Oncology Department, Instituto Valenciano de Oncología, Valencia, Spain; 3 Medical Oncology Department, Vall d’Hebrón Barcelona Hospital Campus, Vall d’Hebron Institute of Oncology (VHIO), Universitat Autònoma de Barcelona, Barcelona, Spain; 4 Department of Biochemistry and Molecular Medicine, Universitat de Barcelona, Barcelona, Spain; 5 Translational Genomics and Targeted Therapeutics in Solid Tumors Group, Medical Oncology Department, Hospital Clinic of Barcelona, IDIBAPS, University of Barcelona, C. Villarroel, 170. 08036 Barcelona, Spain; 6 Medical Oncology Department, Centro de Investigación Biomédica en Red de Cáncer (CIBERONC), Madrid, Spain; 7 Medical Oncology Department, Hospital Universitario Puerta de Hierro, Majadahonda, Madrid, Spain; 8 Apheresis & Cellular Therapy Unit, Department of Hemotherapy and Hemostasis, IDIBAPS, Hospital Clínic, Barcelona, Spain; 9 Endoscopic Unit, Gastrointestinal Service, Hospital Clínic Barcelona, IDIBAPS, CIBERehd, University of Barcelona, Barcelona, Spain; 10 Radiology Department, Hospital Clínic Barcelona, Barcelona, Spain; 11 Pathology Department, Hospital Clínic de Barcelona, Barcelona, Spain; 12 Biostatistics Unit, Faculty of Medicine, Autonomous University of Barcelona, Barcelona, Spain; 13 Barcelona Institute for Molecular Biology, National Science Council (IBMB-CSIC), Barcelona, Spain; 14 Networked Center for Hepatic and Digestive Diseases (CIBER-EHD), Instituto Nacional de La Salud Carlos III, Madrid, Spain; 15 Universidad Peruana Cayetano Heredia, Lima, Peru

**Keywords:** Vaccines, Metabolism, Resistance

## Abstract

**Background:**

Immune check-point blockade (ICB) has shown clinical benefit in mismatch repair-deficient/microsatellite instability high metastatic colorectal cancer (mCRC) but not in mismatch repair-proficient/microsatellite stable patients. Cancer vaccines with autologous dendritic cells (ADC) could be a complementary therapeutic approach to ICB as this combination has the potential to achieve synergistic effects.

**Methods:**

This was a Phase I/II multicentric study with translational sub-studies, to evaluate the safety, pharmacodynamics and anti-tumor effects of Avelumab plus ADC vaccine in heavily pre-treated MSS mCRC patients. Primary objective was to determine the maximum tolerated dose and the efficacy of the combination. The primary end-point was 40% progression-free survival at 6 months with a 2 Simon Stage.

**Results:**

A total of 28 patients were screened and 19 pts were included. Combined therapy was safe and well tolerated. An interim analysis (Simon design first-stage) recommended early termination because only 2/19 (11%) patients were disease free at 6 months. Median PFS was 3.1 months [2.1–5.3 months] and overall survival was 12.2 months [3.2–23.2 months]. Stimulation of immune system was observed in vitro but not clinically. The evaluation of basal RNA-seq noted significant changes between pre and post-therapy liver biopsies related to lipid metabolism and transport, inflammation and oxidative stress pathways.

**Conclusions:**

The combination of Avelumab plus ADC vaccine is safe and well tolerated but exhibited modest clinical activity. Our study describes, for the first-time, a de novo post-therapy metabolic rewiring, that could represent novel immunotherapy-induced tumor vulnerabilities.

**Supplementary Information:**

The online version contains supplementary material available at 10.1007/s00262-022-03283-5.

## Introduction

Immune checkpoint-blockade inhibitors (*ICB*) have shown high activity in MSI colorectal cancer [1–4]. However, this therapeutic approach has limited efficacy in cancers with low tumor mutational burden, such as MSS colorectal cancer, with < 5% best overall response (BOR), 2.2 months median progression-free survival (PFS), 5 months median overall survival and < 20% PFS at 6 months [5].

Immunohistochemical (IHC) analysis of programmed death ligand-1 (PD-L1) as a predictive biomarker has been confounded by multiple unresolved issues that have cast doubt on PD-L1 as an adequate predictive biomarker for *ICB* response. More recently transcriptomic signatures suggest that capturing the complexity of the immune system might be a better strategy to evaluate anti-PD-L1 inhibitor efficacy [6–8]. Our group has recently discovered an immune-metabolic-signature (IMMETCOLS) that appears to identify, across tumor-types, three distinct Clusters with potential clinical implications [9].

Cancer vaccines could be a complementary therapeutic approach to *ICB*. These vaccines aim to stimulate tumor antigen-specific cytotoxic T lymphocytes that recognize and potentially eliminate cancer cells in an antigen-specific manner and therefore convert immunologically “cold” tumors into “hot” tumors. We have previously published a phase II randomized clinical trial that compared autologous dendritic cells (ADC) vaccination plus best supportive care (BSC) with a median PFS of 2.7 months and 6.2 months median OS vs BSC with a median PFS of 2.3 months and a 4.7 months median OS in pre-treated mCRC patients. Although no statistically differences in survival between both arms were observed, ADC-treated patients generated a tumor-specific immune response and ADC therapy was tolerated well [10]. Recently oncolytic vaccines [11] and nanoparticles with tumor whole-cell lysate [12] combined with PD-L1 blockade has shown CD8 T cell activation and pre-clinical efficacy in colorectal cancer models.

Here we have designed a Phase I/II multicenter trial, with translational sub-studies, to evaluate the safety, pharmacodynamics and anti-tumor effects of Avelumab (anti-PD-L1) plus ADC vaccine in heavily pre-treated MSS mCRC patients.

## Methods

### Study design

This was a single arm Phase I/II multicentric study, with translational sub-study, of Avelumab (anti-PD-L1) plus autologous dendritic cell (ADC) vaccine in mismatch repair-proficient (MSS) metastatic colorectal cancer patients previously treated with at least 2 chemotherapy regimens. The study was conducted by the Grupo Español Multidisciplinar en Cáncer Digestivo (GEMCAD) and was registered on ClinicalTrials.gov (NCT03152565). Merck provided the Avelumab. Subjects underwent follow up visits weekly during the first month of treatment and every 2 weeks thereafter. Tumor response evaluation [through the revised response evaluation criteria in solid tumors (RECIST 1.1)], was assessed every 8 weeks (2 months) until disease progression. Toxicity was recorded on every visit using last version of NCI-CTCAE-V 4.03criteria.

Eligibility for inclusion included patients aged ≥ 18, histologically diagnosed MSS colorectal adenocarcinoma, Eastern Cooperative Oncology Group (ECOG) performance status (PS) 0 or 1, measurable disease by RECIST.1.1 criteria and a maximum lactate dehydrogenase level (LDH) level of > 1.5 upper limit normal (ULN) (ULN < 234 U/ml) centrally evaluated at Hospital Clínic Barcelona. Standard parameters for adequate liver, hematological and renal function were mandatory. Patients were required to show progression of metastatic cancer after receiving at least two chemotherapy regimens, with or without targeted therapies. All patients enrolled had signed an informed consent document approved by the investigator’s Institutional Review Board (IRB)/Independent Ethics Committee (IEC). The exclusion criteria included presence of brain metastases, prior organ transplantation, presence of clinical ascites, a modified Charlson index (except for cancer) score > 2, significant infections (acute or chronic), active autoimmune diseases, pregnancy, lactation, positive serological determination of HIV, HBV or HCV, unwillingness to use effective contraception during the trial, a history of other tumors.

This 3 + 3 phase I-II dose de-escalation trial was open to patients with advanced metastatic MSS colorectal cancer patients. Level 1: Avelumab 10 mg/kg biweekly until disease progression or unacceptable toxicity plus 10 × 10^6^ cells of ADC vaccines biweekly for 5 infusions followed by up to 6 infusions every 6 months. If none of the first 3 patients experienced a dose limiting toxicity (DLT) this dose was recommended for phase 2. If 1/3 patients experienced a DLT, 3 more patients were recruited. If < 2/6 limiting toxicities observed, this dose will be recommended for phase 2. If 2/3 or 2/6 patients experienced a DLT, cohort -1 was opened. Level -1: Avelumab 3 mg/kg biweekly until disease progression or unacceptable toxicity + 10 × 10^6^ ADC vaccines biweekly for 5 infusions followed by up to 6 infusions every 6 months. Patients received the combination therapy as follows: a dose of intradermal ADC vaccine (10 × 10^6^ cells/dose) at days 1, 14, 28, 42 and 56 (total of 5 doses), and thereafter every 6 months until disease progression (maximum of 6 additional doses) or unacceptable toxicity; Avelumab was administered intravenously at a dose of 10 mg per kilogram of body weight, every 14 days until disease progression or unacceptable toxicity.

For the production of the ADC vaccine (approved by Spanish regulatory agency AEMPS; and NCT01413295, patients underwent aphaeresis to obtain peripheral blood leucocytes (60 mL total volume, > 5 × 10^9^ mononuclear cells). Autologous monocytes were selected by adherence to culture flasks and then differentiated to DCs by culturing adherent monocytes for 7 days in X-VIVO 15 (Lonza, Walkersville, MD, USA) supplemented with 2% autologous serum, 800 U/mL GM-CSF (Miltenyi Biotech) and 500 U/mL IL-4 (Miltenyi Biotec) and then for an additional 24 h in the presence of 20 ng/mL tumor necrosis factor-α (Miltenyi Biotech), 10 ng/mL IL1-β (Miltenyi Biotech), 20 ng/mL IL-6 (Miltenyi Biotech), 1 μg/mL prostaglandin E2 (Dinoprostona; Pfizer, New York, USA), 20 μg/mL poly (I:C) (Hiltonol; Oncovir Inc, Washington DC, USA) and autologous tumor lysate, using good manufacturing practices standard procedures. Maturation was confirmed by immunophenotyping. Release criteria included > 80% CD80 + , CD83 + , CD86 + , HLA-DR + , absence of T lymphocytes (CD3 +) and monocytes (CD14) (less than 15% CD14, CD3 and CD19 positive cells) and negative microbial test (bacterial, fungi, mycoplasma). Tumor biopsies to generate tumor lysates for ADC vaccine were obtained from colonoscopy samples (*n* = 5) or accessible metastases (liver *n* = 10, lung *n* = 2, peritoneum *n* = 1, lymph node *n* = 1), before study entry. To obtain the lysates, tumors were first washed twice for 30 min with RPMI 1640 (Lonza) supplemented with an antibiotic-antimitotic (100 IU/mL penicillin 100 μg/mL streptomycin and 250 ng/mL amphotericin B, Gibco, Life Technologies Limited, UK) and then disrupted with a GentleMACS dissociator device (Miltenyi Biotech, Bergisch Gladbach, Germany) in RPMI 1640, DNAse I (0,1 mg/mL) and collagenase IV (1 mg/mL), followed by freezing/thawing (five cycles), 25 KGy irradiation by cobalt-60, and subsequently cryopreserved at -80^o^ C until needed for the preparation of DCs. Each vial containing 10 × 10^6^ of ADC plus matured DCs. Upregulation of costimulatory molecules was confirmed in mature DCs (data not included) accordingly to stablished parameters.

## Translational studies

Tumor biopsies from primary tumor or metastases in formalin-fixed paraffin-embedded (FFPE) were obtained before study entry, for MSS, *RAS* and *BRAF* status. Then after 2 months of therapy, a new biopsy was done to evaluate pharmacodynamics changes. Tumor biopsies to generate tumor lysates for ADC vaccine were obtained from colonoscopy (*n* = 5) or accessible metastases (liver *n* = 10, lung *n* = 2, peritoneum *n* = 1, lymph node *n* = 1), before study entry. In 6 cases liver biopsies were done in one center at 2 months post-therapy, to evaluate pharmacodynamic changes after combined therapy. The amount of biopsy obtained in each patient was 0.4 mm^3^. 50 mL of peripheral blood samples (40 mL for PBMCs isolation and 10 mL for serum determination of cytokines) were collected initially and at 2 months to evaluate the immune response.

### Tumor specific T cell response

To determine the effect of the combination therapy in the immune response against tumor, the presence of the tumor-specific T cells was analyzed by cell proliferation, before and after treatment, using the autologous tumor mixed leucocyte reaction (ATMLR) on available patients (*n* = 7). The supernatant of ATLMR was also analyzed to check the presence of inflammatory (IFN-γ) and anti-inflammatory (IL-10) cytokines. Autologous DCs and PBMCs (days 0 and 56) of each patient were thawed and co-cultured (5 × 10^3^ DCs and 1 × 10^5^ PBMCs, triplicates of each condition) in 0.2 ml X-VIVO 15 (Lonza) in 96-well round-bottom culture plates (Nunc, Roskilde, Denmark). To evaluate the synergy of the combination therapy, DCs (pulsed with and without autologous tumor lysate) and avelumab effect on PBMCs was assessed individually and jointly. Plates were incubated in a humid atmosphere of 5% CO2 at 37 °C for 7 days. Eighteen hours before termination of culture, supernatant was harvested for cytokine analysis and each well received 0.5 mCi of [methyl-3H] thymidine at 2 Ci/mmol (TRA310; Amersham Biosciences, Little Chalfont, UK). Uptake of thymidine into DNA was determined using a cell harvester (Perkin Elmer, Boston, MA, USA) involving filtration of lysed cells onto 96-well filter plates (PHDTM cell harvester; Cambridge Tec, Cambridge, MA, USA) and addition of scintillation to each well followed by counting in a TopCount. Levels of cytokines on co-cultured supernatants were analyzed using enzyme-linked immunoabsorbent assay (ELISA) kits from Invitrogen ThermoFisher Scientific, following manufacturer instructions.

### Flow cytometry

Immune phenotyping was performed in available patients (*n* = 12), on PBMCs from samples obtained pre- (day 0) and post- (day 56) treatment by Histopaque gradient centrifugation (Lonza). Staining with anti-human monoclonal antibodies (CD3, CD4, CD8, CD25, CD127, CTLA4, PD1, CCR7, CD62L, CD45RA, CD45RO) was performed and analyzed on an Attune Nxt Flow Cytometer (Invitrogen, Thermofisher Scientific). Details of the flow cytometry panels are shown in Supplementary Table 1.

### Cytokines

Levels of 16 cytokines in serum samples obtained pre- (day 0) and post- (day 56) treatment were determined on available patients (*n* = 16). Simultaneous measurement of Stromal cell-derived factor 1, (CXCL12), interleukin 2 (IL-2), interleukin 6 (IL-6), interleukin 10 (IL-10), interleukin 17A (IL-17a), interleukin 23 (IL-23), interferon gamma (IFNγ), monocyte chemoattractant protein 1 (MCP1, also known as chemokine (C–C motif) ligand 2-CCL2), Fas ligand (FasL or CD95L), matrix metallopeptidase 9 (MMP-9), regulated on activation, normal T cell expressed and secreted (RANTES, also known as Chemokine (C–C motif) ligand 5-CCL5) and vascular endothelial growth factor A (VEGF-A) was analyzed using the ProcartaPlex assay with Luminex™ xMAP technology (Invitrogen, ThermoFisher Scientific) according to the manufacturer’s instructions. Concentrations of vascular endothelial growth factor B (VEGF-B), vascular endothelial growth factor C (VEGF-C), transforming growth factor β 1 (TGFβ1) and transforming growth factor β-3 (TGFβ3) were monitored using enzyme-linked immunoabsorbent assay (ELISA) kits from Wuhan Fine Biotech CO., Ltd and Invitrogen ThermoFisher Scientific.

### RNA-seq: immune-metabolic signatures

RNA was isolated from tumor biopsies in 20 of 25 cases. 4 of 19 cases could not be analyzed basally because of poor RNA quality. Six patients were analyzed after 2 months of therapy, only 5 patients were informative for both biopsies. Quality control was performed with the FASTQ software. Adapter sequences were removed with FASTP. The STAR package was used to map to sequences to the GRCh38 assembly from Ensembl and obtain the number of reads per gene. Gene expression was normalized using the variance stabilizing transformation implemented into the DESeq2 package for R. We used the normalized gene expression data to analyze two signatures. First, a previously published T cell inflamed gene expression profile (GEP) was used with a cut-off below the top tertile of data [13]. Second, a neural network was applied to infer the enrichment of the experimental data in an in-house generated 10-gene signature (IMMECOLS) that discriminates patients across tumor types into 3 distinct immune-metabolic Clusters. [9].

### Efficacy assessments

Following the baseline assessment, subsequent tumor assessments according to RECIST were performed systematically every 8 weeks (± 1 week) using CT scan, physical examination and PCR, albumin, CEA and LDH blood analysis, until disease progression relative to the date of inclusion. RECIST 1.1 criteria were used to assess patient response to treatment by determining progression-free survival (PFS) times, categorization objective tumor response as: complete response (CR), partial response (PR), stable disease (SD), progression of disease (PD), hyper-progressive disease (HPD) and not evaluable (NE). Due to the lack of consensus regarding HPD criteria, and considering the criteria described by previous authors [14] our multidisciplinary team defined HPD with the 4 following criteria: (a) progressive disease at first evaluation (8 weeks after inclusion); (b) increase of > 50% of size in target lesions between baseline and first radiological evaluation; (c) LDH levels increment > 2ULN; (d) decrease in ECOG PS > 2 (e.g., from ECOG PS 0 to ECOG PS > 2 or from ECOG PS 1 to PS > 3) during the first 8 weeks of treatment. Patients who fulfilled at least three of the aforementioned criteria were defined as exhibiting HPD, while patients who accomplish less than three criteria were considered as PD.

### Statistical analysis

We planned to recruit 33 patients to detect an increase of 20% on 6-month PFS. Because an interim analysis recommended early termination for failing to reach the primary endpoint increasing % of PFS, a total of 19 patients were treated with Avelumab and ADC vaccine. PFS and OS were analyzed by Kaplan–Meier curves and compared with a stratified log-rank test. We fitted Cox regression modeling for OS and PFS. Analysis was performed with the R statistical software. For comparisons, unpaired Student’s t-tests or Mann–Whitney U tests were performed. Calculations were made using the Prism software (Graph Pad Software, La Jolla, CA, USA).

## Results

### Patient characteristics

Nineteen patients were enrolled and treated between April 2018 and January 2019 at 5 Spanish centers. Demographic data are listed in Supplementary Table 1. Of 28 patients screened, 9 were not eligible for the study (3 due to poor ECOG PS, 4 due to LDH > 1.5ULN, 1 due to brain metastases in the screening period and 1 because aphaeresis was not feasible). The median number of prior therapies was 3 (range 2–5). *Median time since metastatic diagnoses to study entry was 41.7 months, range (18.4–83 months).* All patients had previously received irinotecan, oxaliplatin and fluoropyrimidines, 14 (74%) had received antiangiogenic agents (11 patients Bevacizumab and 3 patients Aflibercept), 10 (53%) anti-EGFR agents (6 Panitumumab and 4 Cetuximab), 2 patients Regorafenib and 2 patients TAS 102. Genomic and transcriptomic baseline characteristics are listed in Table 1.

**Table 1:** Patient biologic characteristics and treatment efficacy

Patient	RAS	BRAF	GEP	IMMETCOLS	BOR	Survival (months)
01–001	Wild-type	Wild-type	Not evaluated	Not evaluated	SD	DOD (19)
01–003	Wild-type	V600E	High	3	**HPD**	**DOD (4)**
01–004	Wild-type	Wild-type	Low	3	**HPD**	**DOD (4)**
01–007	Wild-type	Wild-type	High	3	PD	DOD (15)
01–008	Wild-type	Wild-type	Not evaluated	Not evaluated	SD	AWD (23 +)
01–009	G12V	Wild-type	Low	3	PD	DOD (18)
01–011	Wild-type	Wild-type	Low	3	SD	DOD (10)
01–012	Wild-type	Wild-type	High	1	PD	DOD (20)
02–002	G12V	Wild-type	Low	1	PD	DOD (17)
02–003	Mutant*	Wild-type	Low	3	**HPD**	**DOD (5)**
02–005	G12V	Wild-type	Low	3	PD	DOD (12)
04–001	Wild-type	Wild-type	High	3	PD	DOD (14)
05–001	G12A	Wild-type	High	3	PD	DOD (11)
05–002	Wild-type	Wild-type	Low	1	**HPD**	**DOD (9)**
05–004	G13D	Wild-type	Low	3	PD	DOD (21)
05–005	G12A	Wild-type	Not evaluated	Not evaluated	PD	DOD (9)
05–006	G12D	Wild-type	Not evaluated	Not evaluated	SD	DOD (15)
09–001	G12S	Wild-type	High	3	PD	DOD (6)
09–002	Mutant*	Wild-type	Low	3	NE	DOD (3)

Bold values indicate patients with hyper-progressive disease

*HPD* Hyper-progressive disease

*not specified RAS mutation

### Safety

Median time from the apheresis and first dose of DC vaccine was 14 days (standard deviation 7.8). Patients did not receive oncologic therapy in the meantime. Three patients were entered in phase I at dose level 1. Because there were no dose-limiting toxicities in the first 3 patients Avelumab (standard dose) was administered with ADC in the rest of patients. The treatment administration was well tolerated, with no grade 3–4 toxicities. The most frequent adverse events were fatigue, diarrhea and flu-like symptoms (see Table 2).

**Table 2:** Adverse events, associated with treatment with avelumab and ADCV

Type of toxicity	Grade 1–2 (%)	Grade 3–4 (%)
Fatigue	3 (16)	1 (5)
Fever	3 (16)	0 (0)
Vomiting	3 (16)	1 (5)
Anorexia	3 (16)	0 (0)
Diarrhea	2 (10)	1 (5)
Arthralgia	2 (10)	0 (0)
Pruritus	2 (10)	0 (0)
Rash	1 (5)	0 (0)
Flu-like symptoms	1 (5)	0 (0)
Anemia	1 (5)	0 (0)
Increased AST	1 (5)	0 (0)
Increased ALT	1 (5)	0 (0)
Hypothyroidism	1 (5)	0 (0)
Stomatitis	1 (5)	0 (0)
Myalgia	1 (5)	0 (0)
Pneumonitis	1 (5)	0 (0)

### Efficacy

One patient was not restaged due to rapidly clinical deterioration. Eighteen patients were evaluable for treatment response. No objective responses were observed. Four patients achieved stable disease (22%), four (22%) experienced hyper progressive disease (HPD) and ten (56%) had progressive disease. The median progression-free survival was 3.1 months [2.1 – 5.3 months] and overall survival was 12.2 months [3.2 – 23.2 months]. An interim analysis (Simon design first-stage) recommended early study termination, because 2/19 patients (11%) were disease free at 6 months. Thirteen patients received additional treatments after disease progression.

### Translational studies

#### In vitro* study*

An increased of tumor-specific T cell proliferation was observed in post-treatment samples after the co-culture of PBMCs with DCs pulsed lysate but not with the PBMCS and the anti-PD-L1 therapy alone. (Supplementary Fig. 6). Levels of inflammatory cytokines (IFN-γ) were also increased after treatment. No increased levels of anti-inflammatory (IL-10) cytokines were observed (Supplementary Fig. 7).

#### Cytokine monitoring

**Fig. 1:**
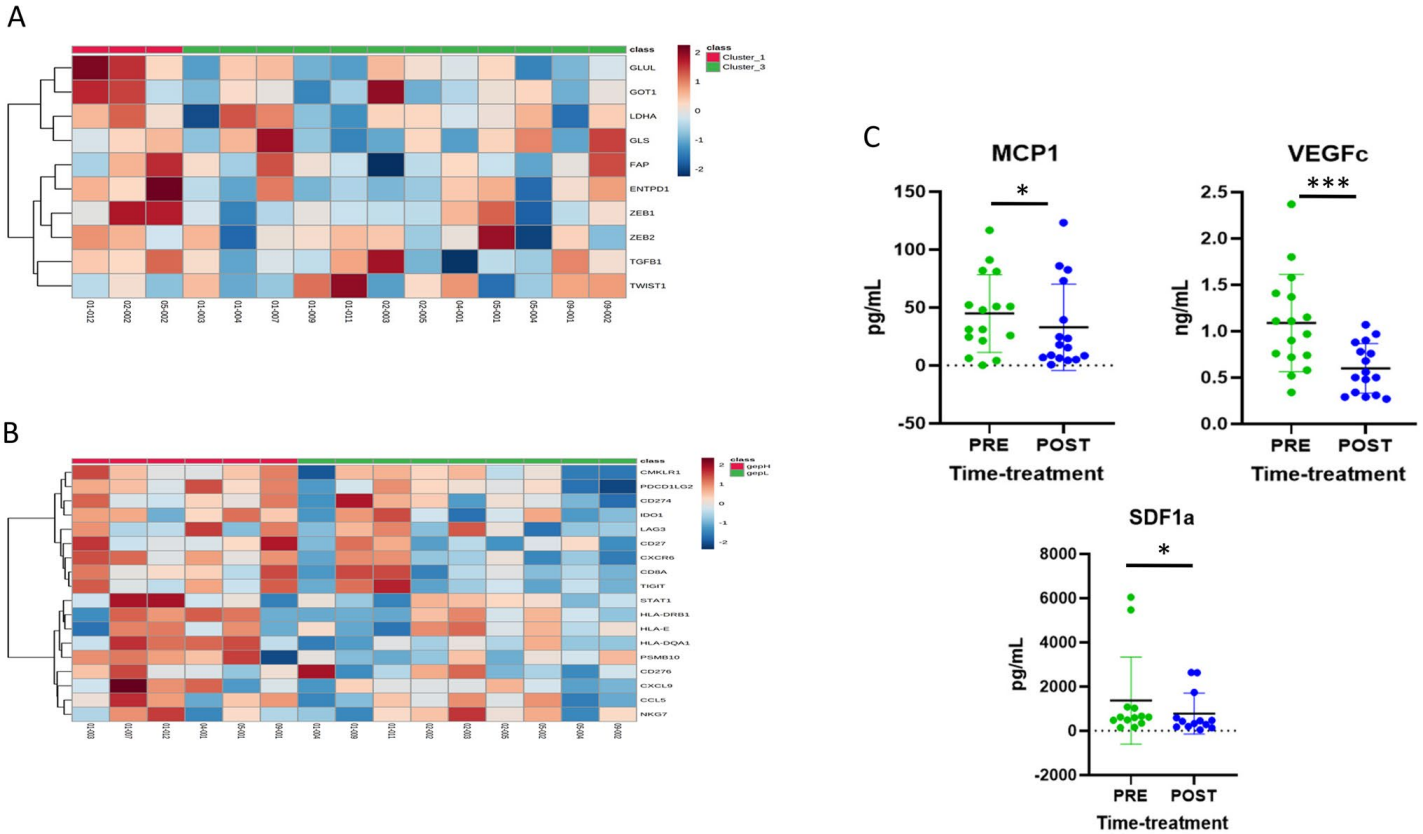
1A. Immune-metabolic signature (IMMETCOLS) baseline expression. 1B. Gene expression pro-immune signature (GEP) baseline expression 1C. Changes in concentrations of cytokines comparing each patient baseline serum with that obtained at day 56 after

The changes in concentrations of 7 of the cytokines analyzed comparing each patient baseline serum with that obtained at day 56 after treatment are illustrated in Supplementary Figs. 1, 2 and 3 and Fig. 1c.

#### Lymphocytic populations

To monitor immune activity in peripheral blood, we analyzed by flow cytometry different lymphocyte subpopulations in the PBMCs of 11 patients before and 56 days after receiving the combination therapy. Proportions of (CD62L^+^CCR7^+^CD45RA^+^CD45RO^−^), effector (CD62L^−^CCR7^−^CD45RA^+^CD45RO^−^), stem memory (CD62L^+^CCR7^+^CD45RA^+^CD45RO^+^), central memory (CD62L^+^CCR7^+^CD45RA^−^CD45RO^+^), effector memory (CD62L^−^CCR7^−^CD45RA^−^CD45RO^+^) and regulatory (CD4^+^CD25^++^CD127^−^) T cells were evaluated, as well as the expression of PD1 and CTLA-4 on CD4 and CD8 T cells. This analysis did not reveal any evidence for significant variations in T cell subsets (Supplementary Figs. 4 and 5). We did not find correlations between different clinical outcomes and the immune subpopulations in peripheral blood (data not shown).

#### RNA immune-signatures

**Fig. 2:**
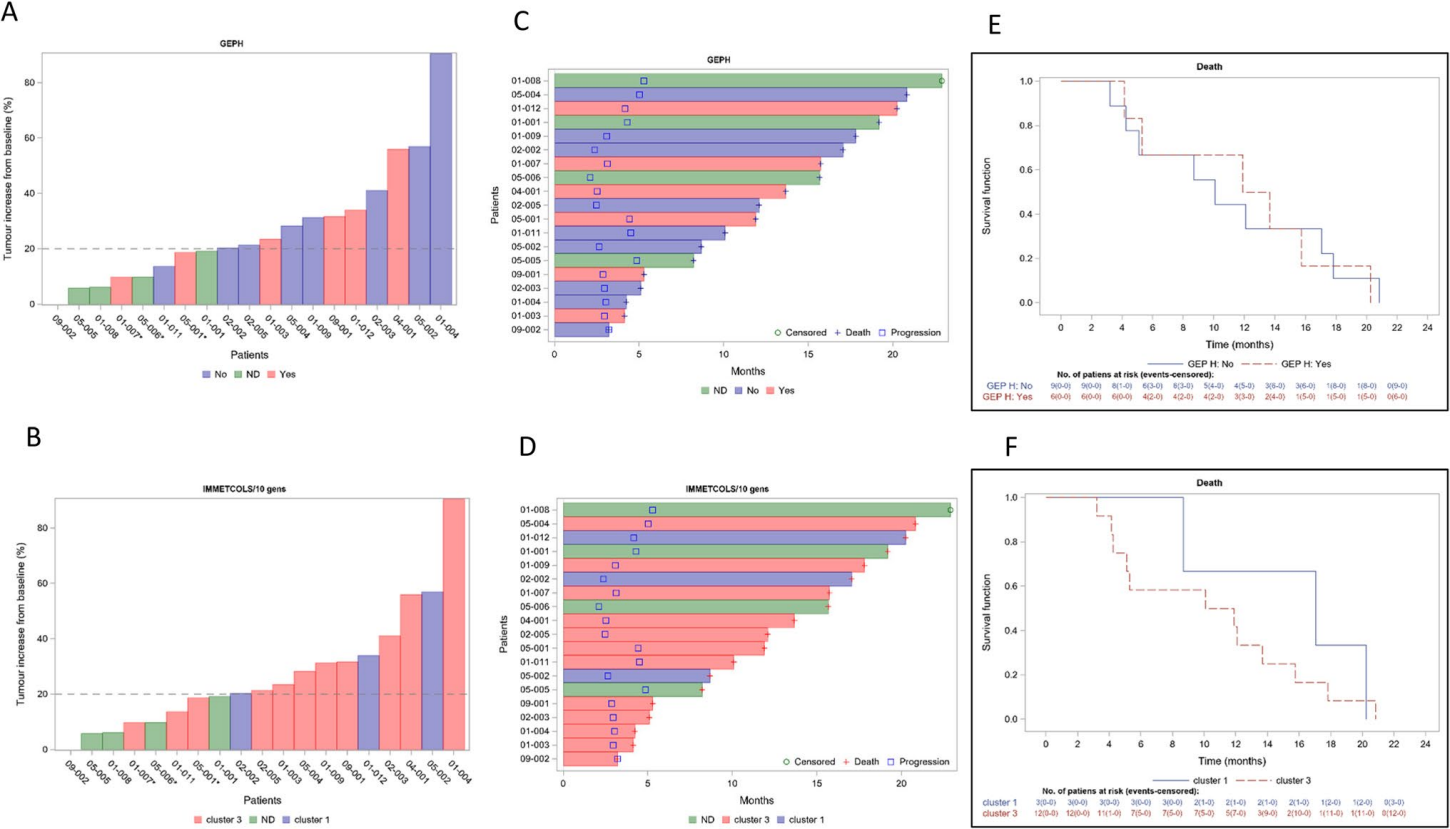
Correlation of both signatures GEP and IMMETCOLS with response rate (2A and 2B), progression free survival or overall survival (2C, 2D). 2E and 2F. Kaplan–Meier curves for overall survival with GEP and IMMETCOLs signatures

To evaluate if treatment efficacy was related to previously published pro-immune signatures (GEP) or to our recently published immune-metabolic signature (IMMETCOLS), we analyzed the baseline expression of both signatures (Fig. 1a and b). The IMMETCOLS signature classifies patients into 3 distinct metabolic Clusters and was cross-validated in the training set. Cluster 1 tumors show enhanced glycolysis, hexosamine biosynthesis pathway, macropinocytosis and branched chain ketoacids (BCKA) synthesis. Cluster 1 is enriched in fibroblast and EMT markers, pro-immune signatures (GEP, PD-L1 and PD1) and also exhausted CD8 + T cells. Concomitant up-regulation of HIF-1a and specific isoforms of enzymes/transporters, suggest that the observed metabolic fingerprint may be mediated by a hypoxic and glutamine deprived tumor microenvironment with a high content of immune infiltrates that facilitate the metabolic cross-talk with cancer cells. Cluster 2 has enhanced glutamine/BCKA oxidation and gain of gluconeogenic/glycogenic ability which are needed for glucose-independent survival and up-regulated enzymes in lipids b-oxidation and glutamine synthesis. Finally, Cluster 3 is characterized by up-regulation of SLC1A5 and SLC7A5 that promotes cancer cell dependence on glucose and increases the need of cytosolic NADPH sustained by concomitant up-regulation of G6PD. Its metabolic signature also suggests the up-regulation of proline, one-carbon metabolism and key players of malate-aspartate shuttle, suggestive of a gain of reductive carboxylation ability. Of 15 patients suitable for analysis, 12 (80%) were assigned to IMMETCOLS Cluster 3 and 3 patients to Cluster 1 (20%). No patient was assigned to Cluster 2. This distribution differs from that found in untreated patients (35% in Cluster 1, 15% in Cluster 2 and 50% in Cluster 3) we also evaluated association of both signatures with response rate, PFS or OS. No significant correlation was found between these signatures and each of these end-points (Fig. 2).

**Fig. 3:**
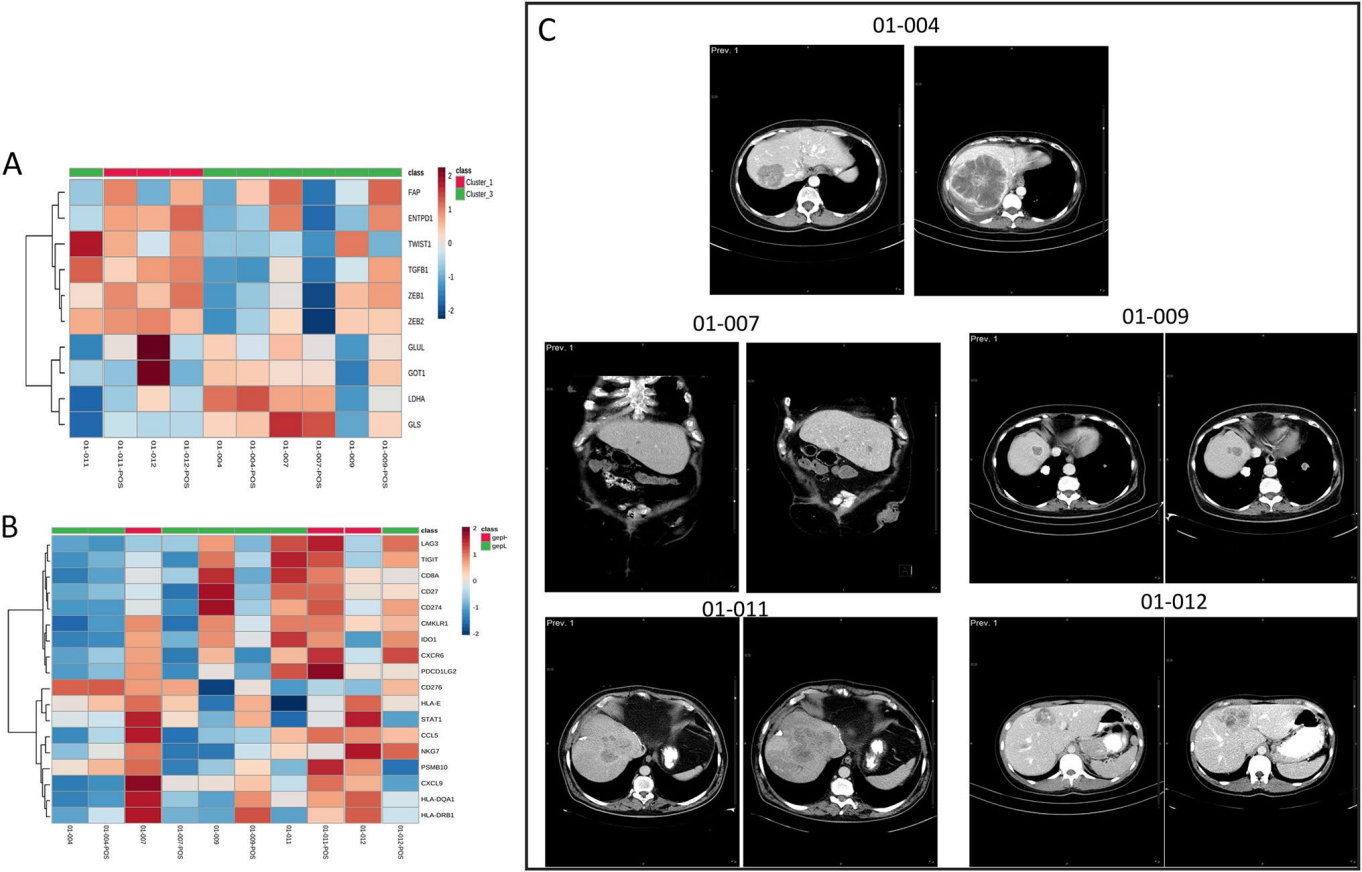
Liver biopsies before and after therapy (2-month evaluation) analyzed in both signatures. 3A. Changes in gene expression with IMMETCOLS signature. 3B. Changes in gene expression with GEP signature. 3C. Radiological CT basally and at 2 month therapy

**Fig. 4:**
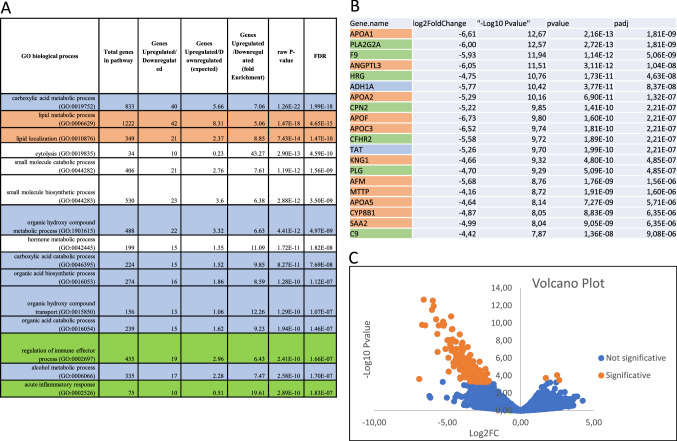
A total of 143 genes significantly changed between pre and post-therapy samples. 4A. More critically GO biological processes changed before and after therapy. 4B. Top 20 genes down-regulated in post-therapy samples compared with pre-therapy samples. 4C. Volcano plot

In order to analyze treatment-associated shifts in both gene signatures (Fig. 3), we also evaluated tumor liver biopsies before and after therapy in five patients. Two patients (01–007 and 01–012) shifted their GEP signature from GEP_H_ to GEP_L_, and one patient from GEP_L_ to GEP_H_ (01–11). In the IMMETCOLS signature analysis, one patient (01–011) converted from Cluster 3 to Cluster 1. A total of 143 genes significantly changed their expression levels between pre and post-therapy samples (Fig. 4 and Supplementary Table 2). The majority of genes (*n* = 140) were down-regulated in post-therapy samples compared with pre-therapy samples. Among biological processes critically up or down-regulated were those related to lipid metabolism and transport (APO1A, ANGPTL3, ADH1, APOA2, APOF, APOC3, RBP4, MTTP, APOA5, ABBC2, SLC27A5, LRP4), acute inflammatory response and regulation of immune effector process (PLA2G2A, HRG, SERPINC1, CFHR2, CFHR5, PLG, C9, F2, ARG1, CCL16, CPNE7) and carboxylic acid, organic hydroxy and organic acid compound metabolism and transport (ADH1A, TAT, PON1, CES1, CYP2E1, ACSM2A, CDO1, UGT2B4, UGT2B7, PIPOX).

## Discussion

Our clinical trial is the first to our knowledge, that evaluates the safety and efficacy of autologous dendritic cell vaccine loaded with tumor lysate in combination with *ICB* in pre-treated mismatch repair-proficient (MSS) metastatic colorectal cancer patients. From this trial we conclude that this strategy is safe and can be developed in a multicenter setting. Regrettably, our study, as has been previously reported with allogeneic vaccines combined with Pembrolizumab [15], or other recent combinations with avelumab and cetuximab (CAVE) [16] or avelumab plus regorafenib (REGOMUNE) [17] did not achieve pre-planned clinical benefit for phase III development.

Although primary-end point of the study was not reached, we observed a median OS of 12.2 months that compares better than the 5 months remarked for trials using anti-PD-1 as monotherapy [5] or between 10–11 months with other strategies with avelumab with approved drugs in mCRC [5, 16, 17]. *Because, patients with left side tumors live longer, they have more chance to be included in trials like AVEVAC study, that included patients with good ECOG performance status that have received at least 2 lines of therapy.* In addition, the safety profile with the combination of avelumab and DC vaccine do not differ with single agent therapies separately [5, 10]. We cannot rule out, that because we use a stringent inclusion criterium (pe. patients with LDH > 1.5ULN were excluded), intrinsic tumor characteristics instead of treatment efficacy, would influence on survival.

The rational for combining vaccines with *ICB* came from extensive pre-clinical research, that elegantly demonstrated that immunologically cold tumors, generated from the murine colorectal cell lines MC38 and CT26, acquired a hot phenotype after various vaccination approaches [11, 12]. It should be emphasized that these two murine models show a much higher tumor mutational burden (TMB) than MSS colorectal cancer patients or mouse colorectal tumor organoid models [18]. Because of scarcity of clinical data addressing whether vaccines could increase antigen presentation in mCRC, we have taken several approaches to address this important issue with different approaches. First, we did a pre-clinical study that shows that the co-culture of PBMCs with DCs pulsed lysate but not with the PBMCS and the anti-PD-L1 therapy increased T cell proliferation and IFNγ. Second, by analyzing circulating cytokine levels, we have found that, while CCL5, TGFβ and MMP9 did not display significant variations after therapy, CCL2 and SDF1a indeed showed decreased levels. Interestingly, the only long-term survivor patient showed a > 240 fold-increase of CCL5 levels post-therapy. Third, the numbers of peripheral blood CD8 effector T cells and CD8^−^PD1^+^ blood cells did not show significant variations after therapy. Finally, the pro-immune GEP signature did not show significant gains in post-therapy tumor tissue liver samples compared with pre-therapy samples. All these translational data, fail to support at least clinically, a strategy to use ADC vaccines in order to inflame immunological “cold” tumors.

Hyper-progressive disease (HPD) was seen in 4/18 (22%) cases and it was of special concern because we did not observe this type of progression in our previous randomized ADC study [10], in spite of a better clinical profile of patients included in the current study (ECOG PS of 0,1 and without poor biological features (basal LDH < 1.5ULN)). We have seen HPD in IMMETCOLS Cluster 1 patients, whose tumor displays characteristics of a mesenchymal phenotypes (1/3) (33%) but also in patients classified as Cluster 3, with epithelial phenotypes (3/12) (25%). HPD has been extensively described in mesenchymal tumors which are usually enriched with M2- polarized, macrophages and Tregs [19, 20]. Fully humanized monoclonal antibodies such as Avelumab, have been found to exert at least part or their antitumor activity through IgG1 antibody-dependent cell mediated cytotoxicity (ADCC) [21]. However, impaired antibody dependent cellular phagocytosis (ADCP) due to M2 polarization, a recently described mechanism of resistance, probably also contributes to immunosuppression in Cluster 1 [22, 23]. We were surprised that HPD occurred also in Cluster 3 patients, characterized by an epithelial-glycolytic phenotype endowed with a reductive carboxylation ability, with M1-polarized macrophage infiltration.

**Fig. 5:**
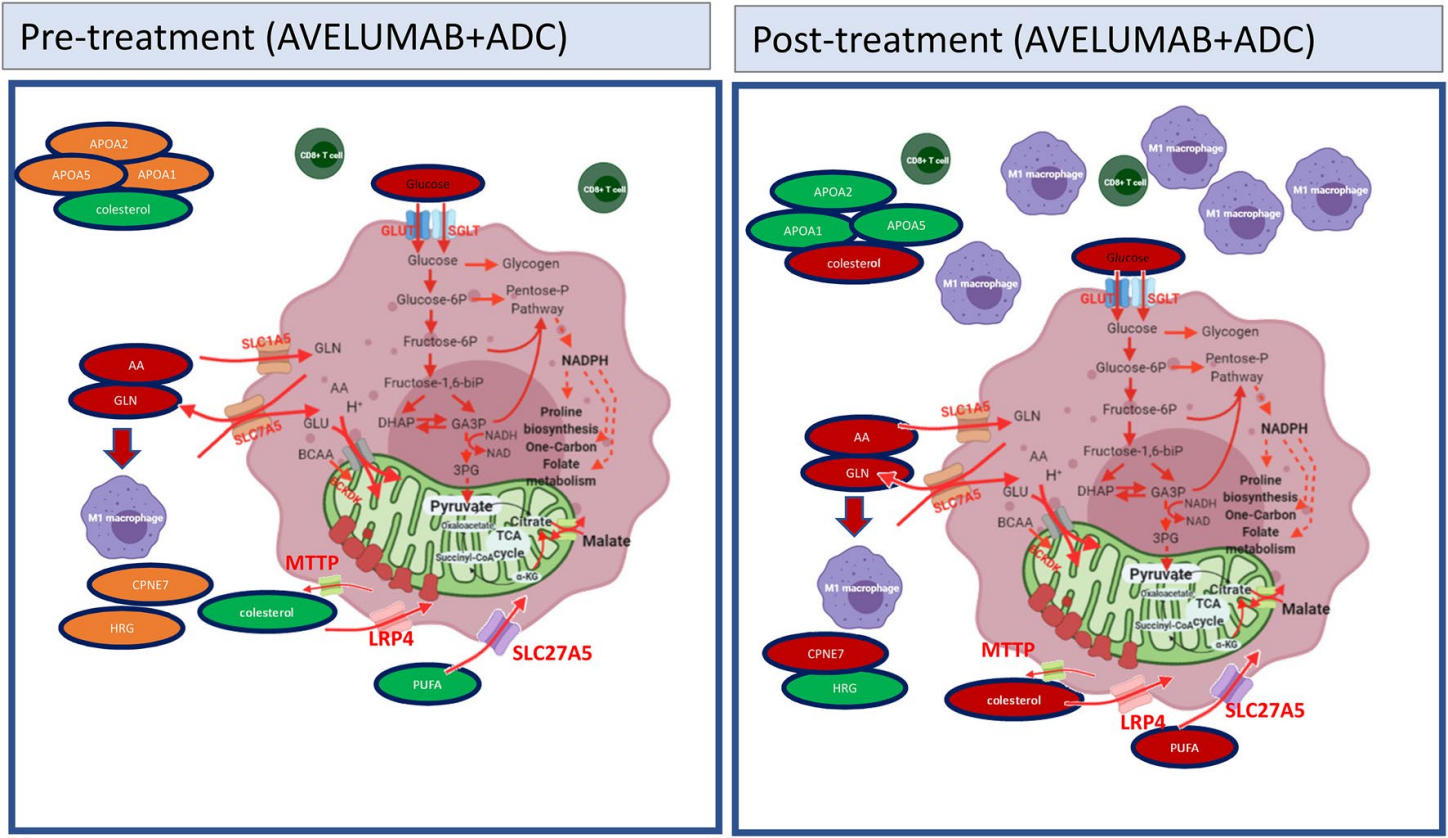
Proposed metabolic rewiring (with lipid consumption instead of only glutamine and glucose feeding). This is the first clinical evidence that this metabolic adaptation is used by colorectal cancer cells, after avelumab and ADC therapy, to boost oxidative stress and contribute to cancer progression. Oxidative stress is compensated in IMMETCOLS cluster 3 tumors with anti-oxidative pathways such as pentose-phosphate pathway

Metabolic competition between cancer cells and tumor microenvironment are tightly intertwined with increased aerobic glycolysis and glutamine consumption [24–26]. Nevertheless, in our analysis of pre- and post-therapy metastatic samples, the most significantly de-regulated genes were related to lipid metabolism and transport. Interestingly, many of the genes down-regulated post-therapy (APOA1, APOH, APOC3, PLG, SERPINC1, C9, ANGPTL3, CFHR5) have been found specifically associated with colorectal liver metastases, suggesting that the observed changes are clinically meaningful [27, 28]. Importantly, some of the lipid metabolism and inflammation genes that we found down-regulated in post-therapy samples play an important role in tumor control and immunomodulation [29–33] and protection from oxidative stress [34, 35]. We speculate that CRC tumors treated with Avelumab and ADC downregulate APOA1 and CCL16, among other genes, but preserve their viability despite increased oxidative stress, thanks to the activation of compensatory reductive pathways (such as pentose phosphate, malic enzyme or isocitrate dehydrogenases). Indeed, these pathways are upregulated in IMMETCOLS Cluster 3. It has been previously described that colorectal liver cancer cells use cholesterol and poly-unsaturated fatty acids to promote ROS and cancer progression [37, 38]. Metabolic rewiring after glutaminase inhibition, with lipid consumption instead of glutamine uptake, has been described pre-clinically [39]. However, to our knowledge, the current study provides the first clinical evidence that a similar metabolic adaptation is also used by colorectal cancer cells after Avelumab and ADC therapy, to boost oxidative stress and contribute to cancer progression (Fig. 5). We propose that a two-pronged metabolic inhibition consisting in [1] to reduce glycolysis [40], glutamine or lipid supplies [41] in order to minimize oxidative stress and [2] or pentose phosphate inhibitors [42, 43] to minimize anti-oxidant defenses, merits consideration in combination with vaccines and *ICB* to achieve clinical efficacy.

We are aware that our study has several limitations. First, the number of recruited patients was small and second, the selected primary end-point (PFS instead of OS) conveys a narrower scope in our observations. Third, the lack of enhanced PFS contrasts with a greater OS observed; this long-term survival effect may be explained due to the limitations of RECIST1.1 interpreting progressive disease in the presence of an immune response. We are also conscious that tumor biopsies are not the most desirable source of antigens in order to prepare a vaccine because of their high heterogenicity compared to synthetic peptides or neoantigens. Finally, the lack of significant post-therapy changes in peripheral blood CD8^+^ effector T cells numbers should not preclude future analyses of these and other immune populations in pre- and post-therapy tumor tissue biopsies. That said, our additional observations of lack of increased GEP scores observed in post-treatment samples suggests that the combined Avelumab plus ADC therapy may fail to boost the cytolytic efficacy of the immune environment in CRCs.

## Conclusion and perspective

We have shown that the combination with Avelumab plus ADC vaccine is safe and well tolerated, although it exhibited modest clinical activity in unselected mCRC patients. Further, we describe that this therapy induces a previously unreported metabolic rewiring after *ICB* and ADC in mCRC which may represent a novel immunotherapy-induced tumor vulnerability. We advocate for new therapeutic strategies, including *ICB* in combination with nanoparticles vaccines or oncolytic virus, to increase antigen presentation along with drugs that target specific metabolic dependences, in selected groups of MSS mCRC patients based on immune-metabolic signatures such as IMMETCOLS, in prospective clinical trials.

## Supplementary Information

Below is the link to the electronic supplementary material.

Supplementary file1 (PDF 50 KB)

Supplementary file2 (PDF 42 KB)

Supplementary file3 (PDF 354 KB)

Supplementary file4 (PDF 33 KB)

Supplementary file5 (PDF 33 KB)

Supplementary file6 (PDF 33 KB)

Supplementary file7 (PDF 45 KB)

Supplementary file8 (PDF 52 KB)

Supplementary file9 (DOCX 15 KB)

Supplementary file10 (DOCX 28 KB)

## Abbreviations

ADC: Autologous dendritic cells
ADCC: Antibody-dependent cell mediated cytotoxicity
ADCP: Antibody dependent cellular phagocytosis
ATMLR: Tumor mixed leucocyte reaction
BOR: Best overall response
BSC: Best supportive care
BCKA: Branched chain ketoacids
CR: Complete response
DLT: Dose limiting toxicity
ECOG: Eastern cooperative oncology group
ELISA: Enzyme-linked immunoabsorbent assay
FasL: Fas ligand
FFPE: Formalin-fixed paraffin-embedded
GEMCAD: Grupo español multidisciplinar en cáncer digestivo
GEP: Gene expression profile
HPD: Hyper-progressive disease
ICB: Immune check-point blockade
IEC: Independent ethics committee
IFNγ: Interferon gamma
IL-2: Interleukin 2
IL-6: Interleukin 6
IL-10: Interleukin 10
IL-17a: Interleukin 17A
IL-23: Interleukin 23
IRB: Institutional review board
LDH: Lactate dehydrogenase level
MCP1: Monocyte chemoattractant protein 1
mCRC: Metastatic colorectal cancer
MMP-9: Matrix metallopeptidase 9
MSS: Mismatch repair-proficient
NE: Not evaluable
OS: Overall survival
PD: Progression of disease
PD-L1: Programmed death ligand-1
PFS: Progression-free survival
PR: Partial response
PS: Performance status
RANTES: Regulated on activation, normal *T* cell expressed and secreted
RECIST 1.1: Response evaluation criteria in solid tumors
SD: Stable disease
SCDF1: Stromal cell-derived factor 1
TGFβ1: Transforming growth factor β 1
TGFβ3: Transforming growth factor β-3
TMB: Tumor mutational burden
ULN: Upper limit normal
VEGF-A: Vascular endothelial growth factor A
VEGF-B: Vascular endothelial growth factor B
VEGF-C: Vascular endothelial growth factor C
