# Supplementary material for: A Phase I-II multicenter trial with Avelumab plus autologous dendritic cell vaccine in pre-treated mismatch repair-proficient (MSS) metastatic colorectal cancer patients; GEMCAD 1602 study
Source: Cancer Immunol Immunother. 2022 Sep 9;72(4):827–40. doi: 10.1007/s00262-022-03283-5 (PMC10025226; doi:10.1007/s00262-022-03283-5)

Suppl Figure 1. Changes in concentrations of 5 of the cytokines analyzed comparing each patient baseline serum with that obtained at day 56 after treatment. Two patients with the mesenchymal subtype (cluster 1) were marked with purple and red colors.

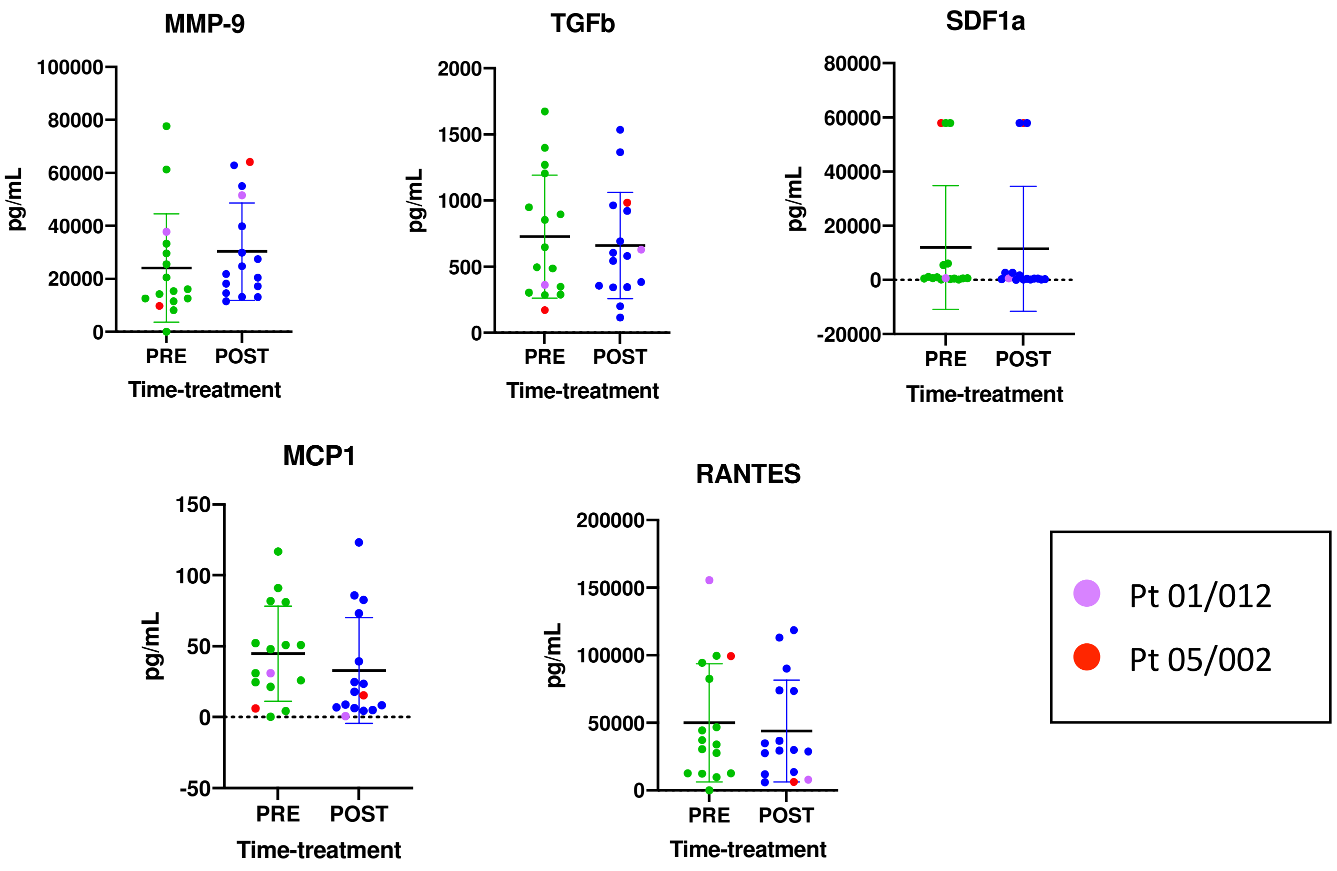

Supplement: Supplementary file 1 — Supplementary file1 (PDF 50 KB) [file 262_2022_3283_MOESM1_ESM.pdf]
