# Supplementary material for: A Phase I-II multicenter trial with Avelumab plus autologous dendritic cell vaccine in pre-treated mismatch repair-proficient (MSS) metastatic colorectal cancer patients; GEMCAD 1602 study
Source: Cancer Immunol Immunother. 2022 Sep 9;72(4):827–40. doi: 10.1007/s00262-022-03283-5 (PMC10025226; doi:10.1007/s00262-022-03283-5)
Supplement: Supplementary file 3 — Supplementary file3 (PDF 354 KB) [file 262_2022_3283_MOESM3_ESM.pdf]

Suppl Figure 3. Changes in concentrations of 5 of the cytokines analyzed comparing each patient baseline serum with that obtained at day 56 after treatment. Two patients with the mesenchymal subtype (cluster 1) were marked with purple.

		SDF1a (CXCL12)		MCP1 (CCL2)		MMP-9		RANTES (CCL5)		TGFb	
		PRE	POST	PRE	POST	PRE	POST	PRE	POST	PRE	POST
cluster 1	01-012	621,13	583,76	31,02	0,80	37756,75	51578,07	155547,57	8139,61	362,00	629,21
	05-002	57900,00	57900,00	6,18	15,39	9768,36	64089,85	99484,76	6436,54	171,86	984,19
cluster 3	01-003	477,43	457,61	24,67	6,95	77600,52	62895,38	94456,90	113016,81	1674,77	923,15
	01-004	624,83	2628,41	21,48	23,41	29637,83	11530,17	46910,55	73501,78	895,84	355,28
	01-007	342,83	180,55	81,04	85,87	16135,28	39849,01	82618,73	27539,78	289,88	343,40
	01-009	598,56	473,17	91,07	73,02	14275,09	27451,65	34099,54	36765,83	304,21	606,33
	01-011	164,30	289,78	81,91	82,55	11574,29	14587,83	44419,39	13687,90	487,50	346,38
	02-003	141,98	45,12	31,10	8,87	12626,31	18201,42	12669,43	12079,40	853,76	963,95
	02-005	6047,88	2637,34	47,90	24,81	15345,96	17236,23	27721,40	34879,56	949,07	1535,72
	04-001	499,16	425,66	0,32	5,14	25542,03	24789,75	12749,64	90208,70	1205,46	1365,20
	05-001	5472,28	1736,62	25,84	17,94	61279,97	13154,55	9889,52	29333,39	349,69	580,46
	05-004	1080,71	198,87	50,86	6,44	12652,06	29846,44	12362,37	30135,08	496,48	201,20
