# Supplementary material for: A Phase I-II multicenter trial with Avelumab plus autologous dendritic cell vaccine in pre-treated mismatch repair-proficient (MSS) metastatic colorectal cancer patients; GEMCAD 1602 study
Source: Cancer Immunol Immunother. 2022 Sep 9;72(4):827–40. doi: 10.1007/s00262-022-03283-5 (PMC10025226; doi:10.1007/s00262-022-03283-5)

Suppl Figure 4. Lymphocyte subpopulations in the PBMCs of 11 patients obtained from peripheral blood before and 56 days after receiving the combination therapy.

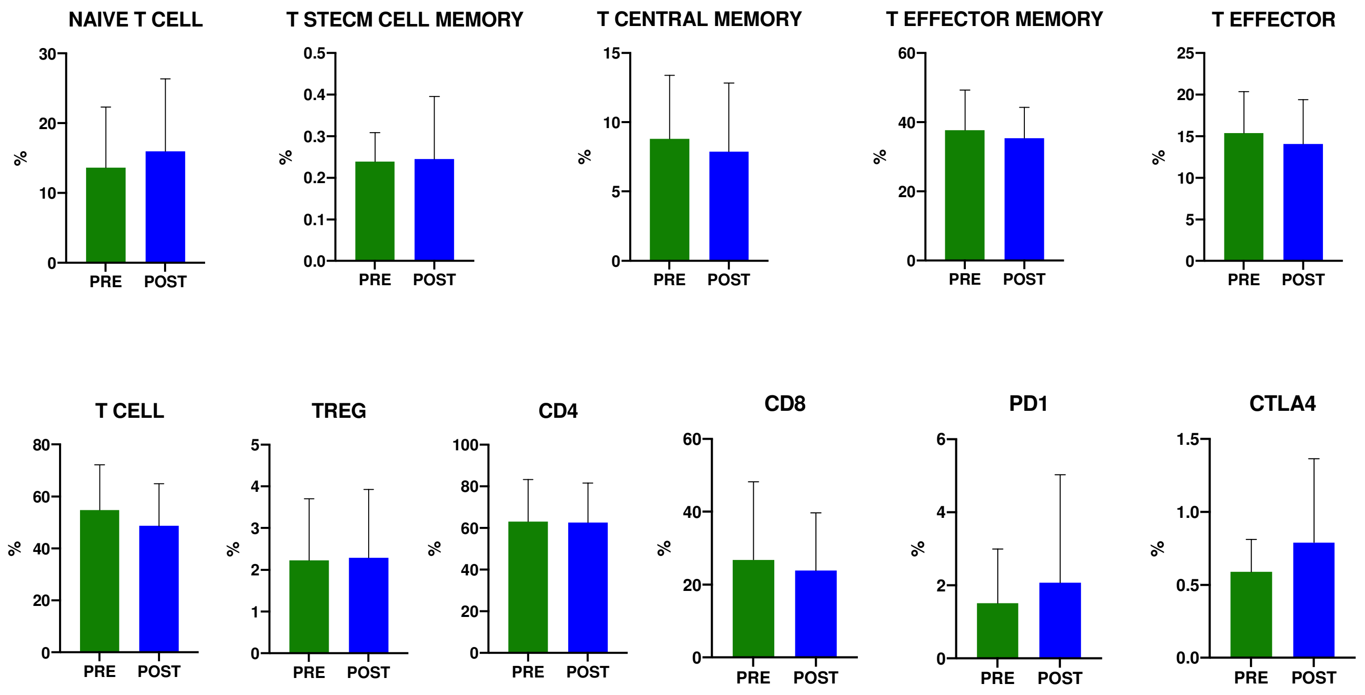

Supplement: Supplementary file 4 — Supplementary file4 (PDF 33 KB) [file 262_2022_3283_MOESM4_ESM.pdf]
