# Supplementary material for: A Phase I-II multicenter trial with Avelumab plus autologous dendritic cell vaccine in pre-treated mismatch repair-proficient (MSS) metastatic colorectal cancer patients; GEMCAD 1602 study
Source: Cancer Immunol Immunother. 2022 Sep 9;72(4):827–40. doi: 10.1007/s00262-022-03283-5 (PMC10025226; doi:10.1007/s00262-022-03283-5)

Suppl figure 6. Autologous tumour mixed leucocyte reaction at day 0 (PRE) and 56 (POST). Results were expressed as counts per minute (cpm). To normalize and analyze all patients together, a proliferation ratio (Cpm [condition] / Cpm [negative control: without tumour lysate and without avelumab]) was calculated. Proliferation was higher after treatment (POST) with the tumour lysate.

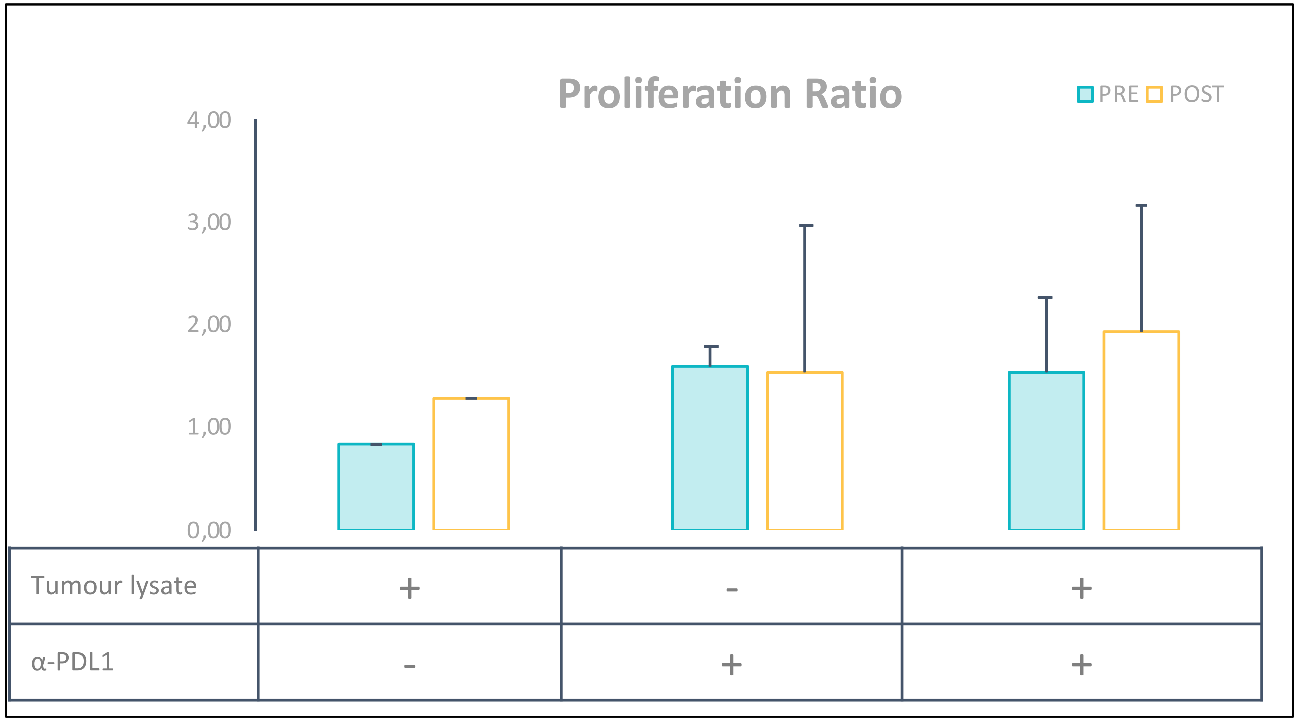

Supplement: Supplementary file 7 — Supplementary file7 (PDF 45 KB) [file 262_2022_3283_MOESM7_ESM.pdf]
