# Supplementary material for: A Phase I-II multicenter trial with Avelumab plus autologous dendritic cell vaccine in pre-treated mismatch repair-proficient (MSS) metastatic colorectal cancer patients; GEMCAD 1602 study
Source: Cancer Immunol Immunother. 2022 Sep 9;72(4):827–40. doi: 10.1007/s00262-022-03283-5 (PMC10025226; doi:10.1007/s00262-022-03283-5)

Suppl figure 7. Cytokine levels of inflammatory (IFN-γ) and anti-inflammatory (IL-10) cytokines of ATMLR supernatants. Levels of IFN-γ were higher on the supernatants of patient's co-cultures after receiving therapy (POST) and no levels of IL-10 were detected (<10 pg/mL).

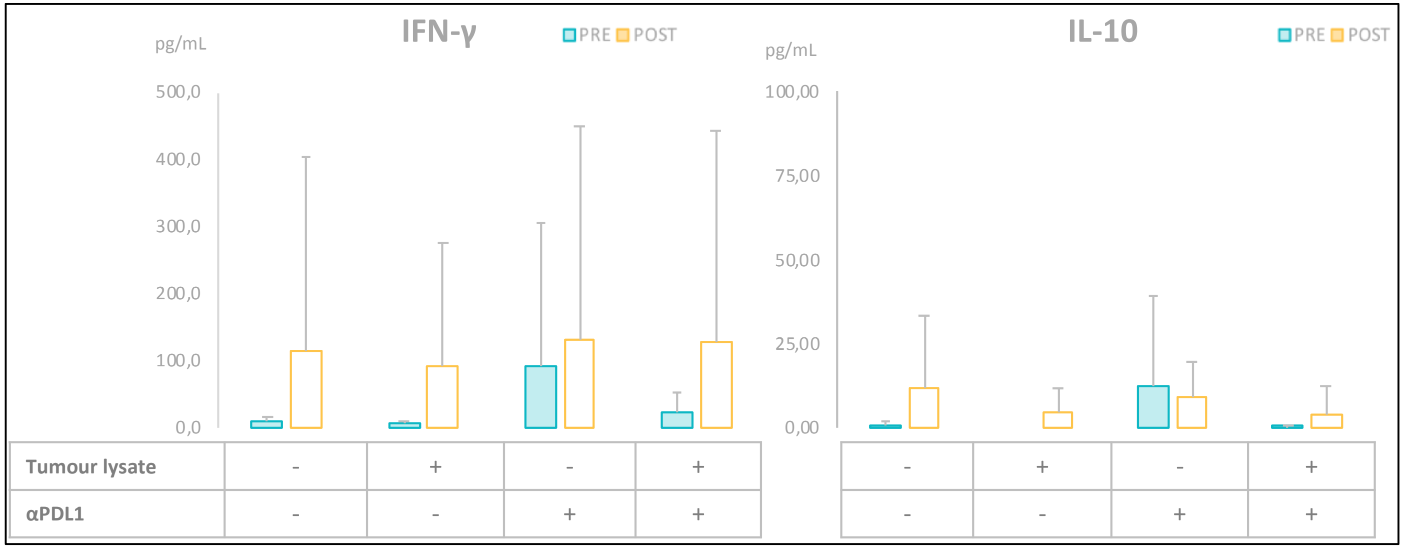

Supplement: Supplementary file 8 — Supplementary file8 (PDF 52 KB) [file 262_2022_3283_MOESM8_ESM.pdf]
