# Supplementary material for: A Phase I-II multicenter trial with Avelumab plus autologous dendritic cell vaccine in pre-treated mismatch repair-proficient (MSS) metastatic colorectal cancer patients; GEMCAD 1602 study
Source: Cancer Immunol Immunother. 2022 Sep 9;72(4):827–40. doi: 10.1007/s00262-022-03283-5 (PMC10025226; doi:10.1007/s00262-022-03283-5)
Supplement: Supplementary file 9 — Supplementary file9 (DOCX 15 KB) [file 262_2022_3283_MOESM9_ESM.docx]

| **Variable** | **Total (n=19), n (%)** |
| --- | --- |
| Median age, (range), years | 62 (32–77) |
| Baseline ECOG PS  0  1 | 13 (68)  6 (32) |
| Gender  Male  Female | 10 (53)  9 (47) |
| Baseline LDH  <234  (234-351) | 11 (58)  8 (42) |
| Primary tumor location  Rectum  Sigmoid  *Descending colon* | 6 (32)  12 (63)  1 (5) |
| Primary tumor resection  No  Yes | 6 (32)  13 (68) |
| *Number of metastatic organs*  1  2  >3 | 4 (21)  7 (37)  8 (26) |
| Stage at diagnosis  II  III  IV | 1 (5)  2 (11)  16 (84) |
| Time from metastatic diagnostic to study entry  <12 months  12-24 months  >24 months | 0 (0)  4 (21)  15 (79) |
| Genotype  BRAF  RAS  *All wild type* | 1 (5)  10 (53)  8 (42) |
| Prior lines of therapies, number  2  3-4  >4 | 3 (16)  11 (58)  5 (26) |
| Liver metastases  Yes | 13 (68) |
| Lung metastases  Yes | 14 (74) |
| Lymph node metastases  Yes | 8 (42) |
| Peritoneal metastases  Yes | 6 (32) |
