# Supplementary material for: A Phase I-II multicenter trial with Avelumab plus autologous dendritic cell vaccine in pre-treated mismatch repair-proficient (MSS) metastatic colorectal cancer patients; GEMCAD 1602 study
Source: Cancer Immunol Immunother. 2022 Sep 9;72(4):827–40. doi: 10.1007/s00262-022-03283-5 (PMC10025226; doi:10.1007/s00262-022-03283-5)
Supplement: Supplementary file 10 — Supplementary file10 (DOCX 28 KB) [file 262_2022_3283_MOESM10_ESM.docx]

| APOA1 | -6,61 | 12,67 | 2,16E-13 | 1,81E-09 |
| --- | --- | --- | --- | --- |
| PLA2G2A | -6,00 | 12,57 | 2,72E-13 | 1,81E-09 |
| F9 | -5,93 | 11,94 | 1,14E-12 | 5,06E-09 |
| ANGPTL3 | -6,05 | 11,51 | 3,11E-12 | 1,04E-08 |
| HRG | -4,75 | 10,76 | 1,73E-11 | 4,63E-08 |
| ADH1A | -5,77 | 10,42 | 3,77E-11 | 8,37E-08 |
| APOA2 | -5,29 | 10,16 | 6,90E-11 | 1,32E-07 |
| CPN2 | -5,22 | 9,85 | 1,41E-10 | 2,21E-07 |
| APOF | -6,73 | 9,80 | 1,60E-10 | 2,21E-07 |
| APOC3 | -6,52 | 9,74 | 1,81E-10 | 2,21E-07 |
| CFHR2 | -5,58 | 9,72 | 1,89E-10 | 2,21E-07 |
| TAT | -5,26 | 9,70 | 1,99E-10 | 2,21E-07 |
| KNG1 | -4,66 | 9,32 | 4,80E-10 | 4,85E-07 |
| PLG | -4,70 | 9,29 | 5,09E-10 | 4,85E-07 |
| AFM | -5,68 | 8,76 | 1,76E-09 | 1,56E-06 |
| MTTP | -4,16 | 8,72 | 1,91E-09 | 1,60E-06 |
| APOA5 | -4,64 | 8,14 | 7,27E-09 | 5,71E-06 |
| CYP8B1 | -4,87 | 8,05 | 8,83E-09 | 6,35E-06 |
| SAA2 | -4,99 | 8,04 | 9,05E-09 | 6,35E-06 |
| C9 | -4,42 | 7,87 | 1,36E-08 | 9,08E-06 |
| ACSM2B | -3,97 | 7,77 | 1,68E-08 | 1,07E-05 |
| AHSG | -4,80 | 7,54 | 2,88E-08 | 1,68E-05 |
| PON1 | -4,02 | 7,54 | 2,90E-08 | 1,68E-05 |
| TF | -3,61 | 7,27 | 5,42E-08 | 3,01E-05 |
| ADH4 | -4,75 | 7,23 | 5,93E-08 | 3,17E-05 |
| HPR | -4,39 | 7,04 | 9,03E-08 | 4,33E-05 |
| CES1 | -4,11 | 7,04 | 9,08E-08 | 4,33E-05 |
| SULT2A1 | -5,13 | 7,04 | 9,09E-08 | 4,33E-05 |
| SLC22A7 | -3,89 | 7,02 | 9,63E-08 | 4,43E-05 |
| CFHR5 | -4,71 | 6,85 | 1,42E-07 | 6,31E-05 |
| CYP2E1 | -4,00 | 6,71 | 1,95E-07 | 8,18E-05 |
| HAO1 | -5,67 | 6,71 | 1,96E-07 | 8,18E-05 |
| MBL2 | -4,44 | 6,49 | 3,23E-07 | 0,00013061 |
| F13B | -4,04 | 6,46 | 3,47E-07 | 0,00013606 |
| HPX | -4,02 | 6,42 | 3,81E-07 | 0,00014543 |
| ACSM2A | -3,43 | 6,37 | 4,27E-07 | 0,00015828 |
| SERPINC1 | -4,38 | 6,30 | 4,99E-07 | 0,00017564 |
| APOH | -4,22 | 6,30 | 5,00E-07 | 0,00017564 |
| CRHBP | -4,29 | 6,11 | 7,80E-07 | 0,00026369 |
| SLCO1B1 | -3,56 | 6,10 | 7,90E-07 | 0,00026369 |
| ACOT12 | -4,93 | 6,05 | 8,91E-07 | 0,00028779 |
| BHMT | -4,10 | 6,04 | 9,06E-07 | 0,00028779 |
| PGLYRP2 | -3,82 | 5,96 | 1,10E-06 | 0,00033339 |
| INHBC | -4,40 | 5,96 | 1,10E-06 | 0,00033339 |
| UGT2B4 | -3,69 | 5,95 | 1,12E-06 | 0,00033339 |
| SLCO1B3 | -4,11 | 5,72 | 1,91E-06 | 0,00055412 |
| AGXT | -3,43 | 5,68 | 2,11E-06 | 0,00058732 |
| CDO1 | -3,50 | 5,68 | 2,11E-06 | 0,00058732 |
| CFHR1 | -4,49 | 5,64 | 2,27E-06 | 0,00061941 |
| F2 | -3,24 | 5,63 | 2,32E-06 | 0,0006201 |
| SAA1 | -4,02 | 5,61 | 2,48E-06 | 0,00064761 |
| IFI44L | -2,80 | 5,60 | 2,53E-06 | 0,00064866 |
| C8A | -3,59 | 5,56 | 2,73E-06 | 0,00068819 |
| ALDOB | -3,85 | 5,51 | 3,10E-06 | 0,00076609 |
| UGT2B10 | -4,24 | 5,49 | 3,25E-06 | 0,0007773 |
| SERTM2 | -4,59 | 5,49 | 3,26E-06 | 0,0007773 |
| FETUB | -4,16 | 5,44 | 3,61E-06 | 0,00084439 |
| AOX1 | -3,41 | 5,41 | 3,91E-06 | 0,0009005 |
| PZP | -3,31 | 5,34 | 4,52E-06 | 0,00102334 |
| CPS1 | -3,61 | 5,29 | 5,07E-06 | 0,00112864 |
| CYP4A22 | -4,43 | 5,28 | 5,20E-06 | 0,00113134 |
| ARG1 | -4,16 | 5,28 | 5,26E-06 | 0,00113134 |
| CREB3L3 | -3,47 | 5,21 | 6,20E-06 | 0,00129487 |
| HPD | -3,59 | 5,21 | 6,21E-06 | 0,00129487 |
| CYP1A2 | -4,22 | 5,20 | 6,31E-06 | 0,0012957 |
| SLC2A2 | -4,56 | 5,05 | 9,01E-06 | 0,00182181 |
| PRG4 | -3,09 | 5,00 | 9,90E-06 | 0,00194774 |
| ITIH1 | -3,33 | 5,00 | 9,93E-06 | 0,00194774 |
| CFHR4 | -4,12 | 4,98 | 1,06E-05 | 0,00203685 |
| AGXT2 | -3,86 | 4,97 | 1,07E-05 | 0,00203685 |
| UPB1 | -3,46 | 4,97 | 1,08E-05 | 0,00203685 |
| RBP4 | -3,76 | 4,96 | 1,11E-05 | 0,00204893 |
| ADCY1 | -2,40 | 4,77 | 1,68E-05 | 0,00303245 |
| SRD5A2 | -3,65 | 4,72 | 1,91E-05 | 0,00339376 |
| FGL1 | -4,15 | 4,69 | 2,06E-05 | 0,00360912 |
| HGFAC | -2,79 | 4,62 | 2,40E-05 | 0,00416551 |
| SERPINA11 | -3,98 | 4,61 | 2,45E-05 | 0,00419466 |
| SLC38A3 | -2,83 | 4,54 | 2,90E-05 | 0,00489932 |
| CYP2A6 | -3,71 | 4,51 | 3,07E-05 | 0,005116 |
| GLYAT | -3,90 | 4,49 | 3,22E-05 | 0,00529876 |
| LECT2 | -4,91 | 4,46 | 3,47E-05 | 0,00564747 |
| LBP | -3,00 | 4,45 | 3,51E-05 | 0,00564747 |
| TFR2 | -2,76 | 4,42 | 3,82E-05 | 0,00606123 |
| RDH16 | -3,34 | 4,39 | 4,08E-05 | 0,00639865 |
| F11 | -3,78 | 4,37 | 4,25E-05 | 0,0065967 |
| HP | -3,78 | 4,33 | 4,70E-05 | 0,00720666 |
| LPA | -3,29 | 4,31 | 4,88E-05 | 0,00740694 |
| SLC1A2 | -2,29 | 4,24 | 5,73E-05 | 0,00852962 |
| AKR1D1 | -3,18 | 4,24 | 5,75E-05 | 0,00852962 |
| MASP2 | -2,90 | 4,19 | 6,42E-05 | 0,00941744 |
| OIT3 | -2,81 | 4,18 | 6,58E-05 | 0,00954387 |
| C6 | -3,52 | 4,15 | 7,16E-05 | 0,01015753 |
| AQP9 | -3,15 | 4,10 | 7,88E-05 | 0,01106389 |
| HSD17B6 | -3,04 | 4,09 | 8,09E-05 | 0,01123961 |
| SPP2 | -3,29 | 4,08 | 8,25E-05 | 0,01135529 |
| ABCC2 | -2,48 | 4,06 | 8,67E-05 | 0,01180222 |
| PPP1R1B | 2,50 | 4,05 | 8,90E-05 | 0,01198996 |
| SLC17A2 | -3,63 | 4,05 | 9,00E-05 | 0,01200763 |
| SLC13A5 | -2,90 | 4,04 | 9,15E-05 | 0,01208528 |
| ETNPPL | -3,87 | 3,96 | 1,10E-04 | 0,01439205 |
| CCL16 | -3,84 | 3,95 | 1,12E-04 | 0,01448136 |
| GPM6A | -3,37 | 3,94 | 1,15E-04 | 0,01477214 |
| UGT3A1 | -2,87 | 3,93 | 1,16E-04 | 0,01477214 |
| PRODH2 | -3,88 | 3,86 | 1,39E-04 | 0,01737829 |
| SERPIND1 | -3,62 | 3,84 | 1,46E-04 | 0,01800162 |
| SLC27A5 | -2,45 | 3,82 | 1,51E-04 | 0,01847466 |
| HJV | -3,36 | 3,81 | 1,55E-04 | 0,01878025 |
| INHBE | -2,90 | 3,80 | 1,57E-04 | 0,01885274 |
| SMLR1 | -4,12 | 3,80 | 1,58E-04 | 0,01885274 |
| GSTA1 | -3,78 | 3,80 | 1,60E-04 | 0,01890101 |
| MLIP | -3,54 | 3,78 | 1,67E-04 | 0,01948333 |
| SLC25A47 | -3,46 | 3,77 | 1,68E-04 | 0,01948333 |
| DNASE1L3 | -3,10 | 3,77 | 1,69E-04 | 0,01948959 |
| ADRA1A | -2,94 | 3,76 | 1,75E-04 | 0,01992362 |
| ESR1 | -2,63 | 3,75 | 1,77E-04 | 0,02003457 |
| ACSM5 | -2,60 | 3,74 | 1,80E-04 | 0,02018383 |
| SLC22A10 | -3,28 | 3,73 | 1,84E-04 | 0,02033346 |
| LRP4 | 1,73 | 3,73 | 1,84E-04 | 0,02033346 |
| TDO2 | -2,73 | 3,70 | 2,00E-04 | 0,02165024 |
| ABCG8 | -2,64 | 3,69 | 2,04E-04 | 0,02191837 |
| GBP7 | -4,12 | 3,64 | 2,28E-04 | 0,02438954 |
| HAO2 | -3,31 | 3,63 | 2,33E-04 | 0,02468973 |
| UGT2B7 | -3,12 | 3,63 | 2,37E-04 | 0,02486956 |
| AVPR1A | -2,88 | 3,62 | 2,42E-04 | 0,02513636 |
| C8B | -3,03 | 3,61 | 2,44E-04 | 0,02513636 |
| TTR | -6,92 | 3,61 | 2,45E-04 | 0,02513636 |
| KLKB1 | -2,55 | 3,57 | 2,72E-04 | 0,02767464 |
| MAT1A | -3,19 | 3,55 | 2,79E-04 | 0,02819894 |
| FST | -2,63 | 3,49 | 3,21E-04 | 0,03221977 |
| SLC25A18 | -2,65 | 3,49 | 3,24E-04 | 0,03230759 |
| CPNE7 | 2,64 | 3,48 | 3,30E-04 | 0,03257571 |
| NUGGC | -2,67 | 3,48 | 3,34E-04 | 0,03281648 |
| CYP3A4 | -3,11 | 3,44 | 3,61E-04 | 0,03515986 |
| ACSS3 | -2,17 | 3,42 | 3,82E-04 | 0,0369732 |
| RSAD2 | -2,30 | 3,35 | 4,50E-04 | 0,04321316 |
| SLC22A9 | -3,44 | 3,33 | 4,64E-04 | 0,04425079 |
| RELN | -2,23 | 3,32 | 4,81E-04 | 0,04550438 |
| LIFR | -2,52 | 3,31 | 4,86E-04 | 0,04562458 |
| HSD17B13 | -3,13 | 3,31 | 4,92E-04 | 0,04593024 |
| PIPOX | -2,27 | 3,29 | 5,19E-04 | 0,04806276 |
| DPYS | -2,39 | 3,27 | 5,33E-04 | 0,04893509 |
| MT1G | -2,92 | 3,27 | 5,35E-04 | 0,04893509 |
| STAB2 | -2,62 | 3,27 | 5,41E-04 | 0,04907363 |
